# Three Milliliters of Peripheral Blood Is Sufficient for Preparing Liquid Platelet-Rich Fibrin (PRF): An In Vitro Study

**DOI:** 10.3390/bioengineering11030253

**Published:** 2024-03-04

**Authors:** Sarah Al-Maawi, Eva Dohle, Robert Sader, Shahram Ghanaati

**Affiliations:** FORM (Frankfurt Oral Regenerative Medicine) Clinic for Maxillofacial and Plastic Surgery, Goethe University, 60590 Frankfurt am Main, Germany

**Keywords:** PRF, platelet-rich-fibrin, regeneration, 3 mL, LSCC

## Abstract

Platelet-rich fibrin (PRF) has assumed an important role in supporting tissue regeneration in different fields. To date, the standard protocol for liquid PRF requires at least 10 mL of peripheral blood. The present study aimed to analyze the composition, growth factor release, and effects on the cell proliferation of PRF samples produced using 3 mL vs. 10 mL of peripheral blood in vitro. Peripheral venous blood from six healthy donors was used to prepare liquid PRF using either 3 mL or 10 mL tubes. Three different centrifugation protocols were used according to the low-speed centrifugation concept. The cellular distribution was evaluated using immunohistology and automated cell count. ELISA was used to determine the release of different growth factors (EGF, TGF-β1, and PDGF) and interleukin 8 at different time points. Primary human osteoblasts (pOBs) were cultivated for 7 days using PRF-conditioned media acquired from either 3 mL or 10 mL of peripheral blood. The results showed that 3 mL of peripheral blood is sufficient to produce a liquid PRF concentrate similar to that acquired when using 10 mL blood. The concentrations of platelets and leukocytes were comparable regardless of the initial blood volume (3 mL vs. 10 mL). Similarly, the release of growth factors (EGF, TGF-β1, and PDGF) and interleukin 8 was often comparable in both groups over 7 days. The cultivation of pOBs using PRF-conditioned media showed a similar proliferation rate regardless of the initial blood volume. This proliferation rate was also similar to that of pOBs treated with 20% FBS-conditioned media. These findings validated the use of 3 mL of peripheral blood to generate liquid PRF matrices according to the low-speed centrifugation concept, which may open new application fields for research purposes such as in vivo experiments and clinical applications such as pediatric surgery.

## 1. Introduction

Wound healing and tissue regeneration are widely faced challenges in different clinical disciplines [1]. Currently, damaged tissue is substituted using either autologous tissue transplantations (autologous bone or skin) or biomaterials (external bone or skin substitute materials). Autologous tissue transplantation is currently still considered the gold standard [2]. However, some drawbacks such as patient morbidity, tissue size limitations, and donor-side burdens underscore the need for innovative alternatives [3]. Biomaterials of natural origin, such as allogeneic and xenogeneic materials, as well as those of synthetic origins, serve as effective therapeutic options for specific indications [4]. Additionally biodegradable polymers such as polylactic acid (PLA) and polylactic-co-glycolic acid (PLGA), as well as biodegradable bone fixation materials based on polyhydroxy acids (polyglycolide acid (PGA), polylactide (PLLA and PDLA)), have shown promising results in different fields [5]. In this context, some limitations must be considered regarding the regenerative capacity of biomaterials. The characteristics of such biomaterials as acellular and non-vascular external materials allow them to only serve as scaffolds to guide and support the host tissue to initiate the regeneration process [6]. Nevertheless, most clinically established biomaterials do not exhibit sufficient inductive potential to initiate the regeneration process, which exacerbates the need for regenerative cells within the defect [7,8]. Another option is harvesting and cultivating specific autologous cells and transferring them to the same patients during the course of the treatment [9]. This approach is clinically promising but still not routinely applied due to the need for a donor site, as well as technical and methodological challenges. Therefore, an autologous, easy-to-apply, and time-saving system is needed to meet current clinical requirements.

Blood concentrates have been used under many different indications for tissue regeneration and wound healing support for more than a decade [10]. These concentrates are obtained from a patient’s own peripheral blood and processed via centrifugation to obtain a bioactive scaffold. Different blood concentration systems have been introduced into clinical practice in recent decades, including platelet-rich plasma (PRP) and platelet-rich fibrin (PRF), as well as their subgroups [11,12]. PRF is already being applied in various fields of regenerative medicine, showing considerable clinical success [13,14]. Additionally, different preclinical studies have been conducted to understand the regenerative properties of PRF and reported a positive influence on cell viability, proliferation, and differentiation in vitro [15,16,17,18]. Furthermore, several studies of our group have addressed the influence of the applied relative centrifugal force (RCF) during the centrifugation of PRF matrices on PRF components and bioactivity. Ghanaati et al. [19] presented the low-speed centrifugation concept (LSCC) for the first time. This concept allows PRF matrices to be enriched with bioactive components from the peripheral blood by reducing the RCF [19]. Moreover, combining PRF with biomaterials as a method of pre-biologization was shown to be a promising method to enhance the regenerative capacity of biomaterials [20].

To date, the preparation of PRF matrices requires a blood volume of at least 10 mL to fill the blood collection tube and sufficiently prepare PRF without disturbing the centrifugation process. In a recent study, we introduced blood collection tubes with a reduced volume (3 mL) to enable in vivo research in small rodents. In that study, blood was obtained from Wistar rats and centrifuged according to the LSCC [21]. The results confirmed that the applied RCF had a similar influence to that previously observed using human blood and 10 mL tubes [21]. However, to date, it remains unknown whether PRF matrices that are obtained using a smaller blood volume of 3 mL would have similar proliferative effects to matrices derived from a 10 mL initial blood volume. Therefore, the aim of the present study was to reduce the required initial blood volume for PRF preparation. For this purpose, the present study investigates the composition and growth factor release of PRF matrices produced using a 3 mL initial blood volume according to the LSCC as a proof of concept. Additionally, primary human osteoblasts serve as a model to evaluate the proliferative effect of PRF matrices derived from a 3 mL blood volume compared to those derived from 10 mL tubes.

## 2. Materials and Methods

### 2.1. PRF Preparation

**Figure 1:**
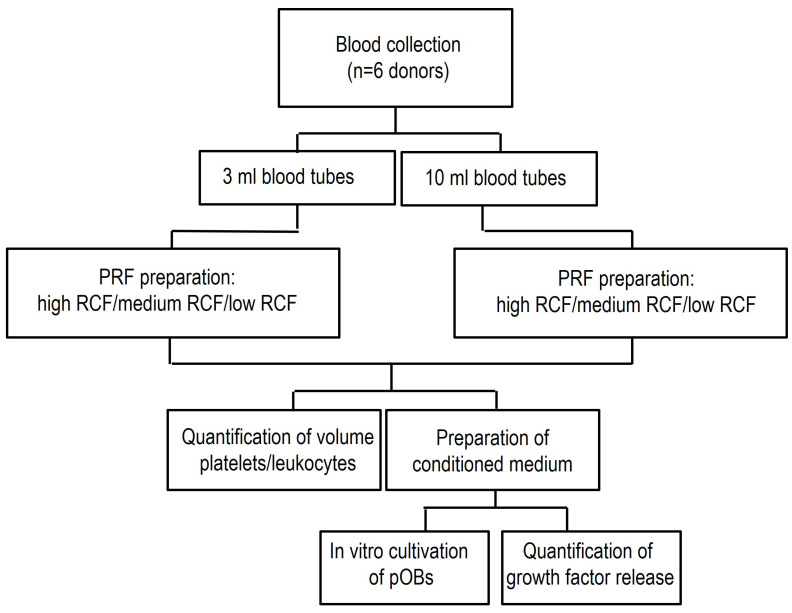
Study design. PRF: platelet-rich fibrin; pOB: primary human osteoblasts.

**Figure 2:**
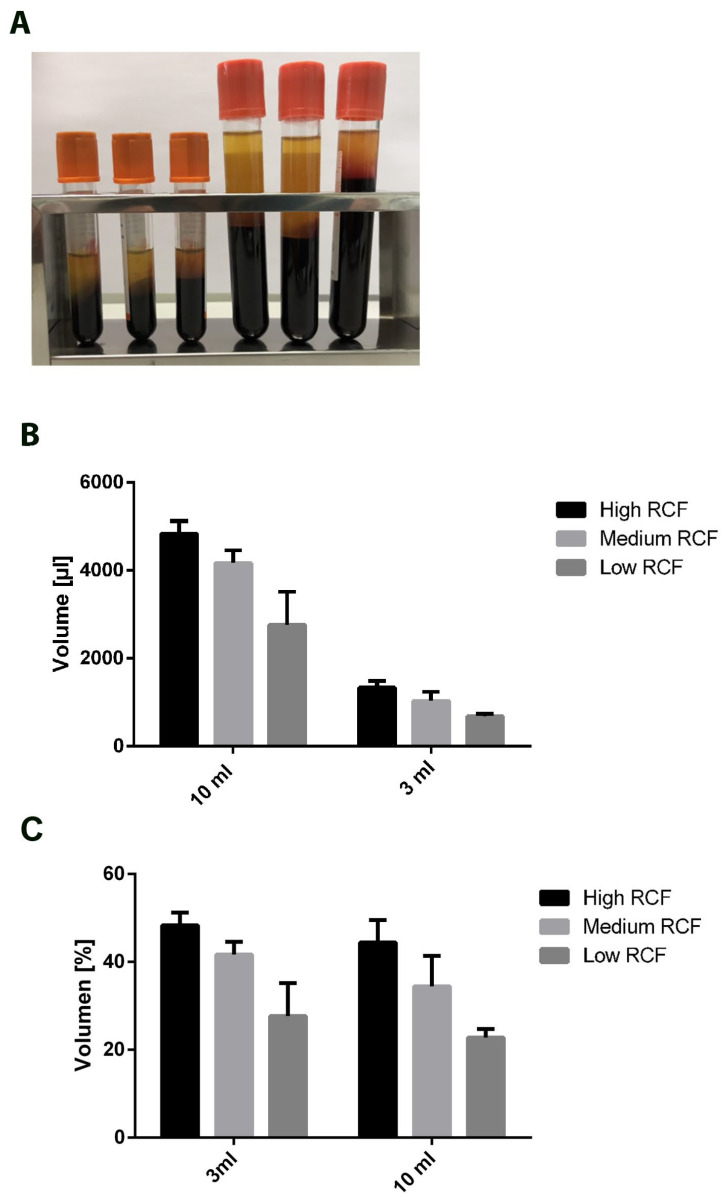
Analysis of the PRF morphology and composition: (**A**) clinical picture of the analyzed PRF samples acquired from the same donor and centrifuged using a high, medium, or low RCF (from left to right) for both 3 mL and 10 mL tubes. (**B**) The determined volume of the acquired PRF samples according to the used volumes and centrifugation protocol. (**C**) Percentage of the PRF volume in relation to the initial blood volume. In the 10 mL group, 100% = 10 mL; in the 3 mL group, 100% = 3 mL.

The application of PRF in this study was approved by the responsible Ethics Commission of Goethe University, State of Hessen, Germany (265/17), in accordance with the principle of informed consent. Peripheral blood samples were collected from 6 healthy donors using a vacuum blood butterfly from the antecubital vein into either 10 mL i-PRF^®^ tubes or 3 mL i-PRF^®^ tubes (Process for PRF, Nice, France) (Figure 1). The donors had to be healthy nonsmokers with no medical history and no use of any anticoagulants. For PRF preparation of the different blood volumes, three different centrifugation protocols were selected. These protocols differed in their relative centrifugal force (RCF), as follows: high-RCF (710 g), medium-RCF (177 g), and low-RCF (44 g) centrifugation protocols. After the centrifugation process, 2 layers could generally be observed regardless of the exerted blood volume and applied RCF: red blood cells at the bottom and injectable PRF at the top (Figure 2A). The liquid PRF was collected from all tubes of each group independently (high RCF, medium RCF, and low RCF), transferred into a syringe, and homogenized. The total volume of each experimental group was calculated before PRF was used for further experimentation. For cell quantification, collected liquid PRF was further processed to carry out an automated cell count. Additionally, liquid PRF from either 10 mL or 3 mL of blood was incubated in an FBS-free DMEM medium (1:1 proportion of PRF: medium volume) on 6-well cell culture plates for 7 days. The supernatants were collected after 1, 2, 5, and 7 days and further used as a conditioned medium (extraction medium) for cell-culture experiments and for protein expression analysis. After 7 days, the PRF samples were fixed for 24 h using 4% Formaldehyde (ROTI^®^Histofix, Carl Roth, Karlsruhe, Germany).

### 2.2. Automated Cell Count

To quantify the cellular components of the PRF acquired from different blood volumes and RCFs, automated cell counting was performed (n = 6 per group) at the German Red Cross Blood Donation Service in Baden-Württemberg-Hessen, Frankfurt am Main, Germany, using a cell counter (HEMAVET, Drew Scientific, Inc., Miami Lakes, FL, USA). The number of platelets within PRF concentrates was calculated as platelets [×10^6^/µL], and the total amount of leukocytes is represented as leukocytes [×1000/µL] in the appropriate figure.

### 2.3. Histological Evaluation

Subsequently, the samples (n = 6) were treated as previously described [17] and embedded in paraffin. Sections of 3–4 µm were then created using a rotary microtome (Leica RM2255; Wetzlar, Germany) and stained using hematoxylin and eosin staining (H&E) for a general evaluation of the cell distribution. Additional sections were stained using specific immunohistochemical antibodies to identify platelets (CD 61) and leukocytes (CD45) using an Autostainer (Lab Vision™ Autostainer 360, Thermo Scientific, Waltham, MA, USA) following previously published methods [4,18]. The CD-61 antibody (1:100 [ab225742]) and CD-45 antibody (1:100 [ab10558]) were used as primary antibodies. The anti-mouse secondary antibody (HRP UltraVision Quanto Detection System; Thermo Fisher Scientific, Waltham, MA, USA) and chromogenic visualization were achieved using AEC peroxidase (Dako). Qualitative histological analysis was performed using a light microscope (Nikon Eclipse 80i; Nikon, Tokyo, Japan) equipped with a Nikon DS-Fi1 digital camera and a Nikon Digital sight unit DS-U3 (Nikon) to capture representative histological images.

### 2.4. Protein Quantification with an Enzyme-Linked Immunosorbent Assay (ELISA)

The content of released growth factors within the PRF culture supernatants (n = 6) derived from either a 3 mL or 10 mL total blood volume was quantified using a quantitative sandwich ELISA after 1 day, 2 days, 5 days, and 7 days of incubation. At each time point of the analysis, culture supernatants were fully removed for protein quantification and replaced with FBS-free DMEM. Briefly, the release of transforming growth factor ß (TGF-β1) and epidermal growth factor (EGF) was quantified using a DuoSet ELISA kit (R&D Systems, Minneapolis, MN, USA) following the instructions from the manufacturer. The absorbance assay was conducted in triplicate using a microplate reader (Infinite M200, Tecan, Grödig, Austria) set to a wavelength of 450 nm with a reference reading at 570 nm. The output data were analyzed using a 4-Parameter Logistic curve fit and presented graphically using Graph-Pad Prism version 6.0 (GraphPad Software, version 10.0.0 for Windows, Boston, MA, USA).

### 2.5. Cell Culture Experiments

Primary cells that were used for this study were obtained from excess tissue and their application was in accordance with the principle of informed consent and approved by the responsible Ethics Commission of the state of Hessen, Germany. Human primary osteoblasts (pOBs) were isolated from cancellous bone fragments, as already described in healthy donors [22] and cultivated in Dulbecco’s Modified Eagle’s Medium Nutrient Mixture F-12 (Sigma-Aldrich, St. Louis, MO, USA) supplemented with 20% FBS (Biochrom, Berlin, Germany) +1% Penicillin/Streptomycin (Sigma-Aldrich, St. Louis, MO, USA) at 37 °C in an atmosphere of 95% air and 5% CO_2_. Cells were passaged in a ratio of 1:2. The cell culture experiments were conducted in triplicate in 96-well plates. To this end, 3.000 pOBs per well were seeded and pre-cultured in DMEM supplemented with 20% FBS. After 24 h, the DMEM was removed, and the cells were further cultivated in the different PRF-conditioned media (PRF in DMEM = the extraction medium) as described in Section 2.1. After 3 and 7 days of cultivation, the cells were analyzed for osteogenic differentiation and proliferation capacity based on the applied extraction medium. The cultivation of pOBs in 20% FBS in DMEM was used for the control experiment.

#### 2.5.1. Calcein AM Staining

Calcein-AM staining of pOBs cultivated in the different PRF-conditioned media was used to determine cell viability with regard to the applied medium. Thereafter, the cell culture medium was removed. Next, the cells were washed twice in PBS and incubated with 5 µM of a Calcein-AM solution (Mobitech GmbH, Göttingen, Deutschland). After 10 min of Calcein-AM incubation, the cells were washed in PBS and subsequently analyzed using a fluorescence microscope (Nikon eclipse TS100, Düsseldorf, Germany).

#### 2.5.2. MTS Assay

To analyze the effects of different PRF extraction media on the proliferation capacity of primary osteoblasts, an MTS assay was performed according to the manufacturer’s instructions. For this test, primary osteoblasts were incubated in the different PRF-conditioned media for 3 days and for 7 days in an atmosphere of 5% CO_2_ and 95% air before being analyzed for cell viability using an MTS CellTiter 96^®^AQ_ueous_ One Solution Cell Proliferation Assay (Promega, Madison, WI, USA) according to the manufacturer’s protocol. For this purpose, the extraction medium was discarded from the cells. After washing the cells with 0.2% HEPES/BSA, we added 120 µL of MTS diluted to a ratio of 1:6 in a fresh medium to the cells, and incubated the mixture for one hour at 37 °C. Next, 100 µL of each sample was transferred to a fresh 96-well cell culture plate, and the absorbance was measured at 492 nm in a microplate reader (GENios plus, TECAN, Crailsheim, Germany). The results were calculated relatively as a percentage of the control.

#### 2.5.3. Alizarin Red Staining and Quantification

To quantify the mineralization in differently treated pOB monocultures, an Osteogenesis Quantitation Kit was used according to the manufacturer’s protocol. Next, the pOBs cultivated in the different PRF-extracted media for 3 and 7 days were washed with PBS and fixed in 3.7% PFA for 15 min at room temperature. After removing the fixative via washing three times with distilled water, 1× alizarin red staining solution was added, and the mixture was incubated at room temperature for at least 20 min. The excess dye was then removed, and the cells were washed four times with deionized water. A solution of 10% acetic acid was added to each well for 30 min. Cells were transferred to a 1.5 mL tube, vortexed, and heated to 85 °C for 10 min. After a centrifugation step of 15 min at 20.000× *g* and neutralizing the pH with 10% ammonium hydroxide, 150 µL of the supernatants was used to quantify alizarin red on a transparent 96-well plate and measured in triplicate at OD_405_ using a microplate reader. The results were calculated relatively as a percentage of the control.

### 2.6. Statistical Analyses

The results are presented as the means ± standard deviation and were evaluated for significant differences at the different time points using a one-way analysis of variance (ANOVA) with the GraphPad Prism 6.0 software (GraphPad Software Inc., La Jolla, CA, USA). Differences were considered statistically significant if the *p* values were * < 0.05 and highly significant if the *p* values were ** < 0.01, and *** < 0.001, and **** < 0.0001. The compiled data were plotted using GraphPad Prism 6.0 software to represent the results graphically. 

## 3. Results

### 3.1. Cellular Composition of the PRF Acquired from Different Blood Volumes and RCFs

The total volume of the different platelet-rich fibrin matrices acquired from different blood volumes (10 mL or 3 mL) and RCFs (high, medium, or low) were determined before further analyzing their cellular compounds. In general, the centrifugation of 10 mL of blood leads to the generation of a higher volume of liquid PRF compared to the results when centrifugating 3 mL of blood, regardless of the applied RCF (Figure 2B). Comparing the amount of PRF based on the individual centrifugation force showed that the highest PRF volume was acquired from blood centrifuged with a high RCF (710 g). The amount of PRF was generally reduced when the RCF decreased, yielding the lowest amount of liquid PRF via centrifugation with a low RCF (44 g). This pattern was obtained for PRF prepared using 10 mL of blood and for that using 3 mL of blood (Figure 2A,B). However, when looking at the percentages of the achieved volumes, similar effects were observed in both groups. Around 50% of the blood volume was acquired as liquid PRF when using a high RCF, regardless of the initial blood volume. The same effect was observed for the other two centrifugation protocols without any statistically significant differences (Figure 2C).

**Figure 3:**
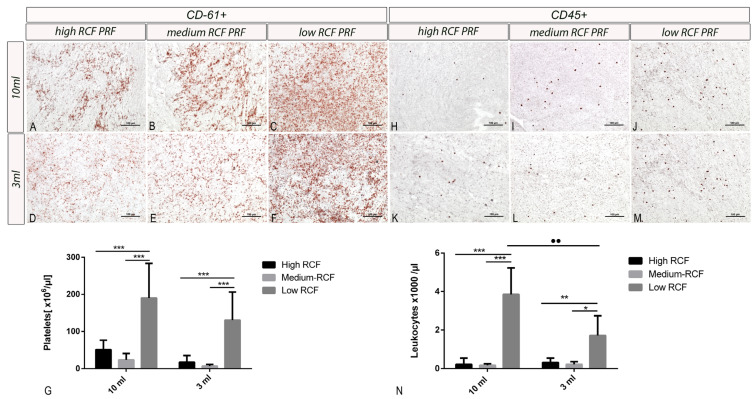
The cellular distribution of (**A**–**F**) platelets (CD-61+) and (**H**–**M**) leukocytes (CD45+) within the different PRF matrices, showing an increased number of cells after decreasing the applied relative centrifugal force (RCF) regardless of the initial blood volume (3 mL vs. 10 mL). (**G**) The platelet concentrations within the acquired PRF samples according to the applied volumes and centrifugation protocol. No statistical analysis was applied. (**N**) The leukocyte concentrations within the acquired PRF samples according to the applied volumes and centrifugation protocol. Differences were considered statistically significant if the *p* values were * < 0.05 and highly significant if the *p* values were **< 0.01, and ***< 0.001 in the intra-individual analysis. For interindividual analysis *p* value was ●●< 0.01.

Histological analysis showed the highest concentration of platelets (CD-61-positive cells) and leukocytes (CD-45 positive cells) in the low RCF centrifugation protocol regardless of the initial blood volume (3 mL vs. 10 mL). A higher RCF resulted in a lower cell concentration. This pattern was reproducible in both the 3 mL and 10 mL groups (Figure 3A–F,H–M). Furthermore, automated cell counting to quantify the cellular components (platelets/leukocytes) of PRF acquired from the different RCFs and blood volumes revealed a significantly higher concentration of platelets and leukocytes when PRF was prepared using the low RCF in both groups of applied blood volume (i.e., 10 mL of blood and 3 mL of blood). In contrast to the concentrations of platelets and leukocytes in the PRF generated via low RCF, the concentrations of platelets in both the high- and medium-generated RCF PRF were considerably and significantly lower. There was no statistically significant difference in the concentration of platelets when comparing the PRF centrifuged with a low RCF using either a 10 mL or 3 mL initial blood volume. However, the PRF centrifuged using a low RCF and a 10 mL blood volume presented a significantly higher concentration of leukocytes compared to that when centrifugating at a low RCF using a 3 mL blood volume (Figure 3G,N). 

### 3.2. Growth Factor Release from PRF Acquired from Different Blood Volumes and Different RCFs

**Figure 4:**
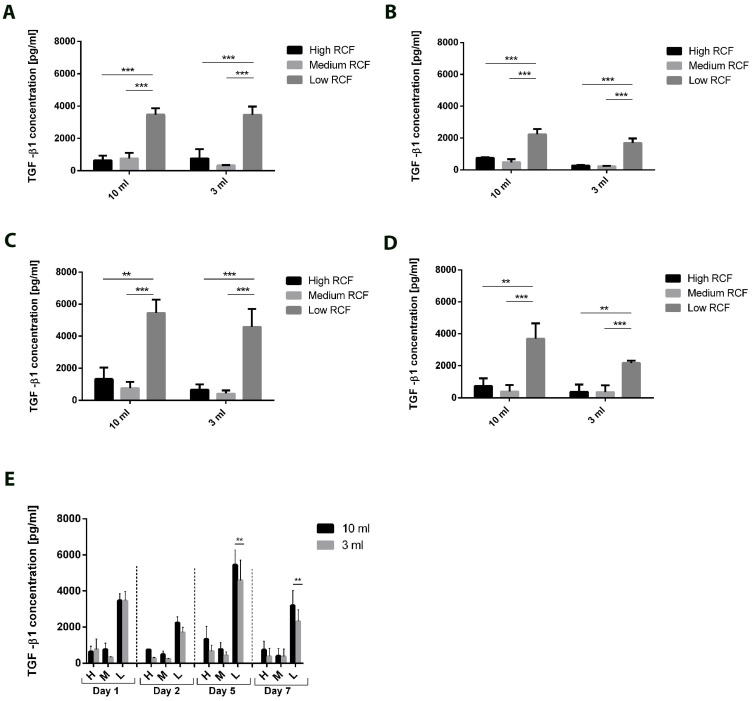
The concentrations of the released TGF-β1 according to the used volumes and centrifugation protocol for Day 1 (**A**), Day 2 (**B**), Day 5 (**C**), and Day 7 (**D**). (**E**) Overview of the released TGF-β1 over 7 days within the analyzed volumes and centrifugation protocol. Differences were considered statistically significant if the *p* values were ** < 0.01 and *** < 0.001.

The growth factor release of transforming growth factor β1 (TGF-β1), epidermal growth factor (EGF), platelet-derived growth factor (PDGF-BB), and interleukin 8 (IL-8) within PRF culture supernatants derived from the different blood volumes and diverse centrifugation forces was quantified at four different cultivation time points (Day 1, Day 2, Day 5, and Day 7). The release of TGF-β1 in the analyzed PRF supernatants clearly differed depending on the applied RCFs, yielding the highest concentration of TGF-β1 when blood was centrifuged with a low RCF (Figure 4A–E). This distinct effect was observed at each analyzed cultivation time point. The concentration of TGF-β1 in the low-RCF PRF culture supernatants was found to be statistically significantly higher than that of lower TGF-β1 concentrations in the medium- and high-RCF PRF supernatants for all tested cultivation time points, regardless of the centrifuged blood volume (Figure 4A–D). The high TGF-β1 concentration in the low-RCF PRF supernatants seemed to be stable over 7 days of cultivation, presenting the highest level on Day 5. However, TGF-β1 was still released in high concentrations on Day 7 (Figure 4D). Furthermore, the TGF-β1 concentrations in supernatants derived from PRF acquired from 3 mL blood were generally similar to those in PRF supernatants acquired from 10 mL blood (Figure 4E). However, a significant difference was observed on Days 5 and 7, when the release of low-RCF PRF was higher after 10 mL of blood was centrifuged compared to the results for a 3 mL blood volume. 

**Figure 5:**
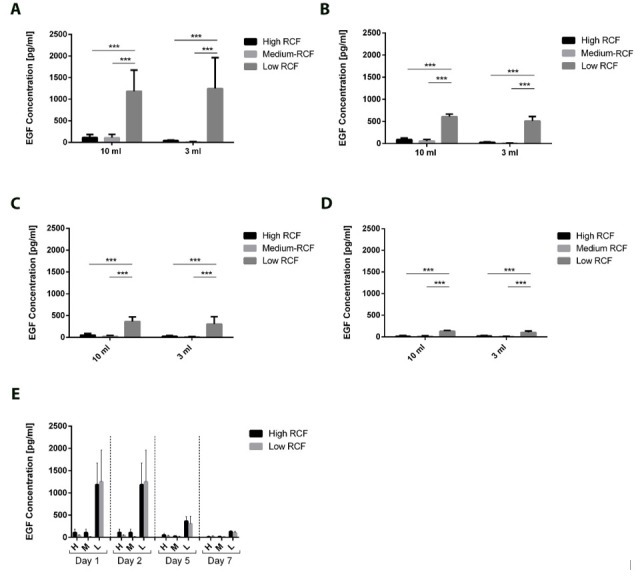
The concentrations of the released EGF according to the applied volumes and centrifugation protocol for Day 1 (**A**), Day 2 (**B**), Day 5 (**C**), and Day 7 (**D**). (**E**) Overview of the released EGF over 7 days within the analyzed volumes and centrifugation protocol. Differences were considered statistically significant if the *p* values were *** < 0.001.

The concentration of EGF in PRF cultivated supernatants was also released in significantly higher quantities from low-RCF PRF than from high- and medium-RCF PRF supernatants at all analyzed cultivation time points, regardless of the centrifuged blood volume (Figure 5A–E). However, EGF released from PRF generated via a low RCF was not stable over the course of PRF cultivation from Day 1 to Day 7 (Figure 5E). While the EGF concentration in the low-RCF PRF supernatants after 1 day could be assessed as >1000 pg/mL (Figure 5A), the EGF concentration in low-RCF PRF supernatants generally decreased over the course of cultivation to a low amount of approximately ~200 on Day 7 (Figure 5D). There were no statistically significant differences between the released concentrations of EGF when using either a 10 mL or 3 mL blood volume when centrifuging using the same RCF.

**Figure 6:**
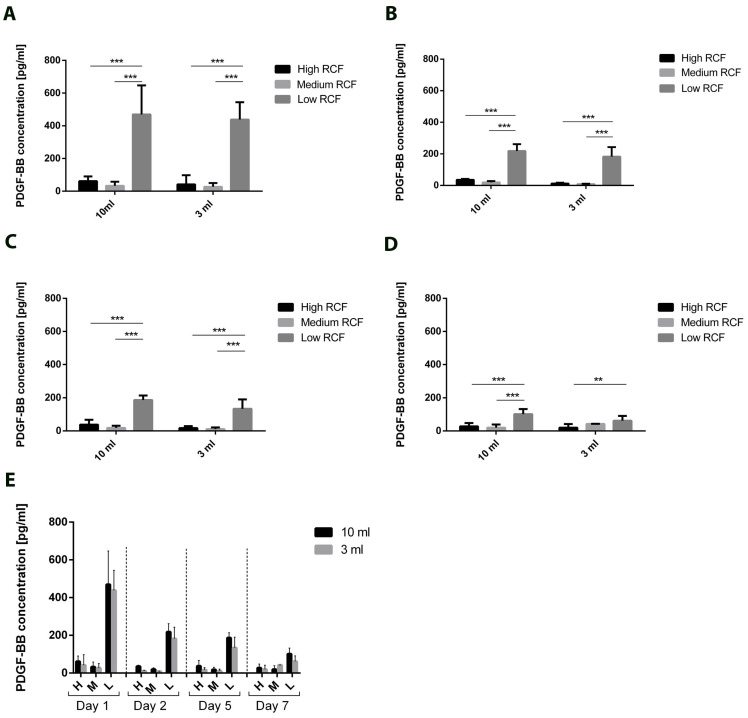
The concentrations of the released PDGF according to the applied volumes and centrifugation protocol for Day 1 (**A**), Day 2 (**B**), Day 5 (**C**), and Day 7 (**D**). (**E**) Overview of the released PDGF over 7 days within the analyzed volumes and centrifugation protocol. Differences were considered statistically significant if the *p* values were ** < 0.01, *** < 0.001.

Similar results were observed for PDGF. In this case, the same pattern of increasing the released concentration by reducing the applied RCF was detected in both the 3 mL and 10 mL groups (Figure 6A–D). The concentrations of PDGF were consistently significantly higher in the groups centrifuged using a low RCF compared to those centrifuged using a medium or high RCF, regardless of the initial blood volume (10 mL or 3 mL). Additionally, the highest released concentration of PDGF was detected on Day 3 and reduced over time. Nevertheless, on Day 7 there remained a detectable concentration of PDGF released from both the 10 mL and 3 mL groups without a statistically significant difference at any time point (Figure 6E).

**Figure 7:**
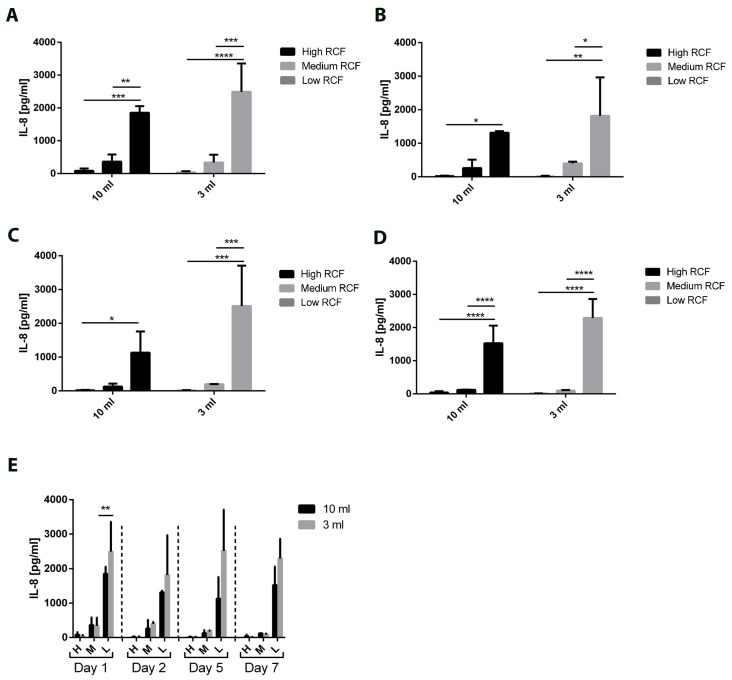
The concentrations of the released interleukin 8 according to the applied volumes and centrifugation protocol for Day 1 (**A**), Day 2 (**B**), Day 5 (**C**), and Day 7 (**D**). (**E**) Overview of the released interleukin 8 over 7 days within the analyzed volumes and centrifugation protocol. Differences were considered statistically significant if the *p* values were * < 0.05 and highly significant if the *p* values were **< 0.01, ***< 0.001, and ****< 0.0001.

Interleukin 8 was detected in all evaluated samples. The highest concentrations were found in the PRF samples centrifuged with a low RCF, regardless of the initial blood volume. These differences were consistently statistically significant (Figure 7A–D). Interestingly, IL-8 showed a constant release pattern throughout the same RCF. However, the results when using a different initial blood volume (i.e., 10 mL or 3 mL) were comparable without any statistically significant difference, except on Day 1 (Figure 7E).

### 3.3. Effect of Different PRF-Conditioned Media on Cell Viability of Primary Osteoblasts

**Figure 8:**
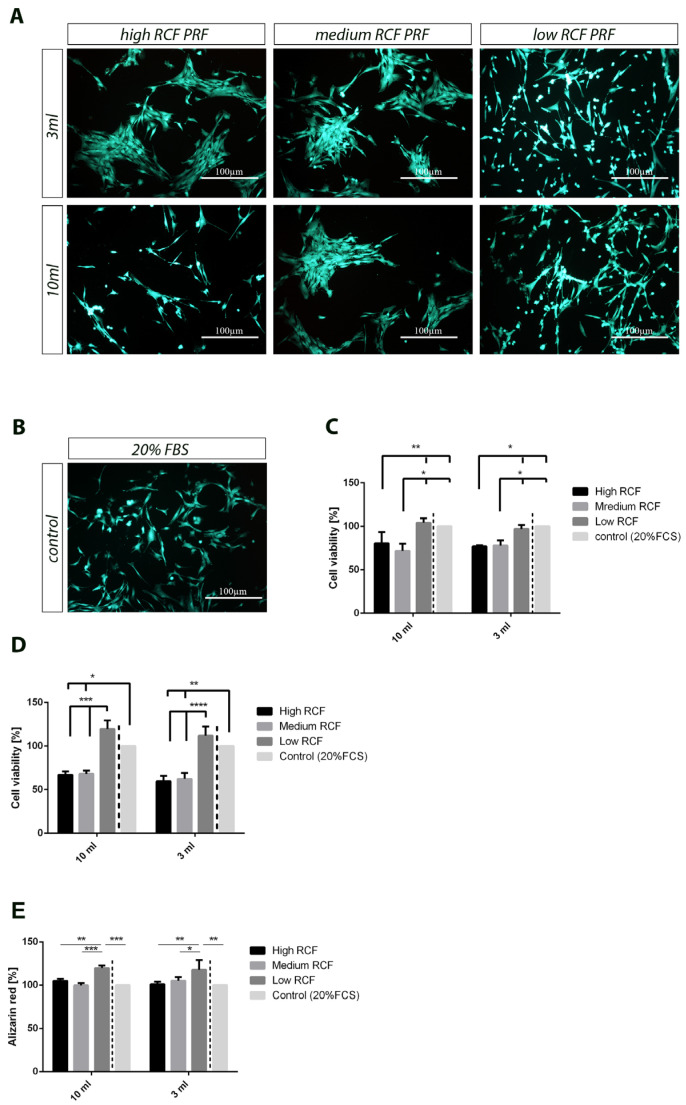
(**A**) Microscopic pictures taken using Calcein-AM staining of primary human osteoblasts (pOBs) treated for 7 days using PRF-conditioned media with either 3 mL or 10 mL of peripheral blood. (**B**) pOBs treated using 20% FBS-conditioned media as a further control group. (**C**) Quantitative results of the proliferation rate (percent of control) of the differently treated pOBs on Day 3 using an MTS assay. (**D**) Quantitative results of the proliferation rate (percent of control) of the differently treated pOBs on Day 7 using an MTS assay. (**E**) Quantitative results of alizarin red staining (percent of control) of the differently treated pOBs on Day 7. Differences were considered statistically significant if the *p* values were * < 0.05 and highly significant if the *p* values were **< 0.01, ***< 0.001, and ****< 0.0001.

The cell viability tests of osteoblasts cultivated in different extraction media harvested from PRF supernatants acquired using differently centrifuged PRFs (high-, medium-, and low-RCF PRF) and different blood volumes (3 mL and 10 mL) revealed higher metabolic activity when osteoblasts were cultivated in a low-RCF PRF-conditioned medium after 3 and 7 days of cultivation compared to the cultivation of osteoblasts in medium- and high-RCF PRF-conditioned media, regardless of the applied blood volume used for PRF preparation (Figure 8C,D). After 3 days of cultivation, the cell viability of primary osteoblasts cultivated in the low-RCF PRF-conditioned medium was comparable to the cell viability of cells cultivated in a control medium supplemented with 20% FBS (Figure 8C). The low-RCF PRF-dependent positive effect on the metabolic activity of primary osteoblasts became even clearer after 7 days of cultivation. At this point, the cell viability of osteoblasts was not only significantly higher in the low-RCF PRF-conditioned media compared to that in the medium- and high-RCF PRF extraction media but was also higher than the cell viability of osteoblasts cultivated in the control medium with 20% FBS (Figure 8D). The Calcein-AM staining of primary osteoblasts cultivated in the different extraction media also confirmed these results. The cell viability assay showed that a higher amount of living cells could be detected when the cells were cultivated in low-RCF PRF-conditioned media compared to the results when using a high- and medium-RCF PRF extraction media (Figure 8A,B).

### 3.4. Mineralization Capacity of pOBs in Response to Different PRF-Conditioned Media

With regard to osteogenic differentiation, we quantified the mineralization of primary osteoblasts to analyze the effects of different PRF-conditioned media using a high, medium, and low RCF with either 3 mL or 10 mL of blood. Cells cultivated in a low-RCF PRF-conditioned medium revealed the highest rate of mineralization estimated via alizarin red staining and quantification compared to cells that were cultivated in medium-RCF PRF-extracted and high-RCF PRF-extracted media (Figure 8E). The calculated ability of primary osteoblasts to mineralize in response to PRF-conditioned media yielded similar results for the different applied blood volumes. Furthermore, the primary osteoblasts cultivated in a low-RCF PRF-conditioned medium revealed an even higher mineralization capacity than that of the control medium, in which cells were supplemented with 20% FBS (Figure 8E). This difference was statistically significant. 

## 4. Discussion

Platelet-rich fibrin (PRF) is a promising autologous blood concentrate system that is being applied in both clinical therapy and scientific research. PRF has been widely used in various regenerative indications, including oral and maxillofacial surgery [23,24], aesthetic medicine [25], and orthopedic treatment [26]. PRF’s regenerative properties and autologous origin make it a favorable tool to support tissue regeneration. In addition to its bioactive capacity to release different growth factors [27,28], PRF was also shown to have an antibiotic effect [29]. Additionally, PRF’s fibrin scaffold allows the integration of different biologically active components and thus serves as a drug delivery system [30]. 

To date, the standard protocol for PRF preparation requires the use of at least 10 mL of peripheral blood to obtain a sufficient result [31,32]. In a clinical setting, especially when treating adults, this blood volume is easy to obtain without any serious burden for the patient. Some large reconstructive interventions may even require up to 10 PRF tubes (i.e., 100 mL of blood). This requirement can be easily managed in the operating theater. However, other procedures such as endodontic treatment, the periodontal treatment of small defects, aesthetic injections, and intraarticular injections of small joints often require only a small amount of PRF. Additionally, harvesting 10 mL of blood or more from young pediatric patients is more complex to perform and may place a higher burden on young patients than on adults. However, younger patients may often profit from the application of this regenerative concept. In addition to clinical applications, the use of PRF for research purposes sometimes requires a small amount of PRF, especially when working in vivo. Therefore, the present study introduced the possibility to reduce the initial blood volume required and generate sufficient PRF matrices using 3 mL blood tubes as an alternative to the standard 10 mL blood volume.

For this purpose, we analyzed the composition, bioactivity, and proliferative effects of PRF generated from 3 mL of peripheral blood. Standard PRF generated from 10 mL of peripheral blood was used as a control. In this context, different RCF applications were tested following the low-speed centrifugation concept (LSCC), as previously described [19]. 

The results showed that it is possible to gain a reasonable amount of PRF using 3 mL of peripheral blood. We observed a dynamic change in the gained PRF volume proportional to the applied RCF. In this way, a higher PRF volume was achieved when a high RCF was applied. This pattern was observed for the PRF matrices prepared using 10 mL and 3 mL alike. Generally, the use of a low RCF resulted in a significantly higher concentration of cells compared to the application of a high RCF. This pattern was also observed in both the 3 mL and 10 mL groups. Interestingly, the portion of the initial blood volume that was turned into PRF was similar regardless of the initial blood volume (3 mL vs. 10 mL). This result was evident when looking at the percentage of PRF volume gained after centrifugation. For example, around 50% of the initial blood volume became liquid PRF after centrifugation, regardless of the initial blood volume (Figure 2C). Analysis of the cellular components including platelets and leukocytes showed comparable platelet concentrations (cell number per microliter) when PRF was prepared using the same RCF and either a 3 mL or 10 mL blood volume without any statistically significant differences. Similarly, the concentrations of leukocytes were comparable in the respective PRF matrices prepared using a high or medium RCF when comparing the 3 mL and 10 mL groups. However, with a low RCF, the leukocyte concentrations were significantly lower when PRF was prepared using 3 mL of peripheral blood compared to 10 mL. These results are primarily a proof of concept for our recently published studies describing the low-speed centrifugation concept [19]. Second, these results showed a reproducible pattern in both tested groups (3 mL vs. 10 mL) and indicated a comparable concentration of cells in both groups. Characterizing the cellular components is a crucial step to validate the quality of the resulting PRF when using a 3 mL initial blood volume instead of 10 mL. 

Similar results were observed when examining the concentrations of the released growth factors. Here, similar concentrations of EGF, TGF-β1, PDGF, and IL-8 were released over 7 days with respect to the applied cultivation ratio (1:1; PRF: cultivation media) when PRF was centrifuged using the same RCF, regardless of the initially used blood amount (3 mL vs. 10 mL). Remarkably, the level of TGF-β1 was significantly higher in the PRF group generated from 10 mL of peripheral blood compared to 3 mL only when a low RCF was applied. One explanation for this observation may be the cell concentrations (i.e., leukocytes and platelets within the respective sample). In this study, we analyzed the release of growth factors over time. Therefore, the quantified growth factors included not only the existing concentrations immediately after centrifugation but also the growth factors actively released by the leukocytes and platelets embedded in the PRF matrix (Figure 3). This hypothesis is strongly supported by the fact that the significant differences observed for TGF-β1 were found during the late timepoints (Days 5 and 7). At these timepoints, the quantified growth factor concentrations were likely produced by leukocytes and platelets. Therefore, the concentrations of the growth factors (Figure 4) correlated with the concentrations of included cells (Figure 2). Consequently, the applied RCF likely has an indirect influence on the concentrations of growth factors by influencing the number of cells producing them. A direct influence of RCF on the growth factors is rather improbable due to their relatively small molecular weight. However, more research is needed to further prove this observation.

The present results are in line with those of a previous study that investigated the quality of PRF acquired from 3 mL of peripheral blood from rats. However, in that study, no comparison with 10 mL tubes of blood was performed [21]. No further studies analyzing PRF from a reduced initial blood volume at this time point were found in the literature, which makes this study the first to introduce this aspect.

After analyzing the cellular components and growth factor release, we further evaluated the proliferative effects of PRF using primary human osteoblasts (pOBs) in vitro. In this step, PRF-conditioned media from 3 mL of peripheral blood was compared to two controls. First, we applied the standard in vitro cultivation procedure using 20% FBS as a supplement, followed by cultivation using PRF-conditioned media with 10 mL of peripheral blood. The results showed that the highest proliferation rate of pOBs was found in the groups treated with the PRF-conditioned media and centrifuged using a low RCF, regardless of the initial blood volume (3 mL vs. 10 mL). Additionally, the results in the low-RCF groups were comparable to those when pOBs were treated with 20% FBS, without any statistically significant differences. Moreover, pOBs treated with PRF centrifuged under a low RCF showed a higher mineralization tendency compared to those in all other groups. In this context, no differences were found when comparing the groups using 3 mL and 10 mL of peripheral blood. These outcomes highlight the functionality and bioactivity of liquid PRF matrices acquired from 3 mL of peripheral blood. However, the time point investigated in this study may be too early to validate the differentiation and mineralization, as the pOBs are still in a state of proliferation. Different studies have outlined the regenerative effects of PRF acquired from 10 mL of peripheral blood on pOBs in vitro [33,34]. These studies are in line with the present results. The capacity of PRF to provide a similar effect to 20% FBS for the in vitro cultivation of pOBs was recently demonstrated [35], further supporting the results of the present study. Our effects observed in vitro are of high value for cell therapy procedures, in which cells are harvested from patients, cultivated in vitro, and reimplanted into the same patient as a type of autologous transplantation [36]. In this procedure, the use of PRF from the same patient instead of external FBS would greatly help keep this autologous system closed without external additives. However, more research is needed to further optimize the use of PRF as a supportive tool for cell-based therapy.

The use of PRF to support tissue regeneration shows promising clinical results. Most studies have evaluated the effects of PRF on the regeneration process for adult patients. For example, PRF was used to support bone regeneration after tooth loss via socket preservation [37] periodontal soft tissue regeneration [38], sinus lift [39], sport medicine [40], and in aesthetic rejuvenation [41]. Although PRF has been widely used for different applications in adults, very few case reports have discussed the supportive effects of blood concentrates in the treatment of pediatric patients [42,43]. This observation may be related to the increased complexity in harvesting peripheral blood from pediatric patients. Therefore, the present results may open new application possibilities for blood concentrates in pediatric surgery by reducing the initially needed blood volume.

On the other hand, PRF generated from 3 mL of peripheral blood may facilitate the application of blood concentrates in animal research when using small animals such as rats or rabbits. When looking at the literature, it becomes very clear that harvesting 10 mL of blood in an autologous setting is nearly impossible when using small rodents. Therefore, different research groups have sought to modify the PRF preparation protocols to reduce the required blood volume for in vivo research [44]. However, to date, there is no standardized protocol. Some studies used 5 mL [34] blood extracted via aortic puncture, while others used only 500 µL via cardiac puncture [45]. In general, these studies focused on the outcome of PRF in the analyzed treatment but did not analyze the composition and bioactivity of the harvested PRF. Therefore, it remains unclear whether the PRF used in this study was functional. Recently, our group introduced the preparation of PRF using 3 mL of peripheral blood via cardiac puncture in small rats (200–250 g) as a terminal procedure. In that study, we evaluated only the components and bioactivity of the harvested PRF [21]. However, no studies have yet analyzed both the characteristics of the harvested PRF and its regenerative capacity in vivo. In this context, the presented 3 mL tubes and preparation protocol could be used as a fully autologous system similar to a clinical setting and applied in vivo without placing a further burden on the animals or requiring the use of donor animals. After standardization and establishment, this step will allow a deeper investigation of the role of PRF for different fields in vivo. However, more research is still needed in this area.

Overall, the present study analyzed the composition, growth factor release, and proliferative effects of PRF in vitro. However, one of the limitations of the present study was its limited dataset when analyzing the in vitro effects of PRF from 3 mL of peripheral blood on pOBs. Further research is needed to establish a protocol for PRF supplements in vitro as an alternative to FBS. Future research could also continue to evaluate PRF matrices in vivo using the presented 3 mL tube system. Moreover, the present study analyzed only liquid PRF produced using 3 mL of peripheral blood. Further research is needed to evaluate whether it is possible to generate well-formed, solid PRF clots using 3 mL tubes. Additionally, PRF is a complex biological construct including a large amount of different signaling molecules, proteins, and cells. In this study, we focused on biological composition by analyzing the concentration of leukocytes and platelets along with studying the morphology and quantifying some growth factors. In this sense, more research is needed to further understand the regenerative effects of PRF and further analyze its components.

Within the limitations of this study, the presented data validated the recently published low-speed centrifugation concept and demonstrated that 3 mL of peripheral blood is sufficient to prepare liquid PRF with a reasonable concentration of leukocytes and platelets, as well as a high regenerative capacity. 

## 5. Conclusions

The present study analyzed the hypothesis of whether the bioactivity of liquid PRF is affected when generated using a reduced initial blood volume of 3 mL instead of 10 mL. The results confirmed that the composition, bioactivity, and in vitro proliferative effects of PRF matrices generated using 3 mL of peripheral blood are comparable to those generated from 10 mL of peripheral blood. These findings validate the use of 3 mL of peripheral blood to generate liquid PRF matrices according to the low-speed centrifugation concept. This possibility may open new fields of clinical application to support regeneration in pediatric surgery. Additionally, the use of PRF as an in vitro supplement provides a further tool to support cell-based tissue regeneration by allowing cell harvesting and cultivation within a fully autologous system. Moreover, the introduction of 3 mL tubes and the validation of their functionality offers an important tool for the standardized evaluation of PRF’s therapeutic capacity in vivo, even when using small rodents.

## Data Availability

The data that support the findings of this study are available from the corresponding author upon reasonable request.
